# Effects of new hypnotic drugs on cognition: A systematic review and network meta-analysis

**DOI:** 10.1016/j.sleepx.2023.100094

**Published:** 2023-11-19

**Authors:** Mengzhen Zhou, Rujia Liu, Jiyou Tang, Shi Tang

**Affiliations:** a Department of Neurology, the First Affiliated Hospital of Shandong First Medical University, Jinan, Shandong, PR China; b Yuhuangding Hospital, No, 20, Yuhuangding East Road, Zhifu District, Yantai, Shandong, PR China; c Department of Neurology, Shandong Provincial Hospital Affiliated to Shandong First Medical University, Jinan, Shandong, PR China

**Keywords:** DORA, Cognition, Insomnia, Dose–response, Meta-analysis

## Abstract

**Background:**

Insomnia is a common disease, and the application of various types of sleeping pills for cognitive impairment is controversial, especially as different doses can lead to different effects. Therefore, it is necessary to evaluate the cognitive impairment caused by different sleeping pills to provide a theoretical basis for guiding clinicians in the selection of medication regimens.

**Objective:**

To evaluate whether various different doses (low, medium and high) of anti-insomnia drugs, such as the dual-orexin receptor antagonist (DORA), zopiclone, eszopiclone and zolpidem, induce cognitive impairment.

**Methods:**

The PubMed, Embase, Scopus, Cochrane Library, and Google Scholar databases were searched from inception to September 20th, 2022 for keywords in randomized controlled trials (RCTs) to evaluate the therapeutic effects of DORA, eszopiclone, zopiclone and zolpidem on sleep and cognitive function. The primary outcomes were indicators related to cognitive characteristics, including scores on the Digit Symbol Substitution Test (DSST) and daytime alertness. The secondary outcomes were the indicators associated with sleep and adverse events. Continuous variables were expressed as the standard mean difference (SMD). Data were obtained through GetData 2.26 and analyzed by Stata v.15.0.

**Results:**

A total of 8702 subjects were included in 29 studies. Eszopiclone_high_ significantly increased the daytime alertness score (SMD = 3.00, 95 % CI: 1.86 to 4.13) compared with the placebo, and eszopiclone_high_ significantly increased the daytime alertness score (SMD = 4.21, 95 % CI: 1.65 to 6.77; SMD = 3.95, 95 % CI: 1.38 to 6.51; SMD = 3.26, 95 % CI: 0.38 to 6.15; and SMD = 3.23, 95 % CI: 0.34 to 6.11) compared with zolpidem_low_, zolpidem_high_, DORA_low_, and eszopiclone_mid_, respectively. Compared with the placebo, zopiclone, zolpidem_mid_, and eszopiclone_high_, DORA significantly increased the TST (SMD = 2.39, 95 % CI: 1.11 to 3.67; SMD = 6.00, 95 % CI: 2.73 to 9.27; SMD = 1.89, 95 % CI: 0.90 to 2.88; and SMD = 1.70, 95 % CI: 0.42 to 2.99, respectively).

**Conclusion:**

We recommend DORA as the best intervention for insomnia because it was highly effective in inducing and maintaining sleep without impairing cognition. Although zolpidem had a more pronounced effect on sleep maintenance, this drug is better for short-term use. Eszopiclone and zopiclone improved sleep, but their cognitive effects have yet to be verified.

## Highlights

• There is a lack of research on Dora and cognition at present, and this article makes up for its shortcomings.
• Animal experiments have proved that Dora can slightly improve cognition, but the clinical evidence is insufficient.
• This paper also involves the safety of Dual orexin receptor antagonists, Zopiclone, Eszopiclone, Zolpidem sleep and sleeping drug treatment.

## 1 Introduction

Since the outbreak of coronavirus disease 2019 (COVID-19), more than 70 million people have developed depression, 90 million have developed anxiety, and hundreds of millions of people have developed insomnia disorders [1,2]. A recent report by the World Health Organization showed that the global incidence rates of anxiety and depression significantly increased (by 25 %) in the first year of the pandemic [3]. Large-scale epidemiological surveys in the USA and the UK also reported proportions of insomnia patients with depressive symptoms of 23 % and 21 %, respectively [4]. Patients with severe anxiety disorders are more prone to depression and even suicidal behavior. Thus, improving sleep and reducing excessive arousal is urgent.

The first generation of hypnotic drugs were barbiturates, which act on the gamma-aminobutyric acid (GABA) system [5]. GABA is an important inhibitory neurotransmitter in the central nervous system that mediates approximately 40 % of inhibitory nerve conduction and plays an important role in control of fear, anxiety, and convulsion-related neuronal hyperactivity [6]. In the 1960s and 1970s, the second generation of sedative and hypnotic drugs—benzodiazepines—was developed. Compared with barbiturates, benzodiazepines had a wider safety range, high safety, and few adverse reactions. However, long-term use produced considerable adverse reactions, leading to drug dependence in nearly 30 % of patients [7,8]. Third-generation nonbenzodiazepine sedatives and hypnotics selectively act on benzodiazepine receptors, exhibiting similar pharmacological characteristics to benzodiazepines [9]. These drugs are characterized by fast sleep onset, increases in deep sleep and prolongation of sleep but do not affect the normal sleep architecture of healthy people and even improve the sleep architecture of patients [10].

At the end of the 20th century, researchers began to develop new sedative hypnotic drugs to reduce adverse reactions in patients. Zopiclone is a third-generation cyclopyrrolone hypnotic agent. This drug is a quick-acting sedative hypnotic agent mainly suitable for use in patients with difficulty sleeping and can shorten sleep latency because of its low likelihood of inducing dependence [11]. Zopiclone is widely used to inhibit GABA receptors (i.e., a GABA receptor antagonist) and benzodiazepine drugs. Zopiclone is the r-isomer; in December 2004, eszopiclone (the s-isomer of this drug) was approved by the FDA for the treatment of insomnia. Eszopiclone is approximately 50 times stronger than zopiclone and has a significantly longer half-life. Compared to zopiclone, eszopiclone is better tolerated, less likely to lead to drug dependence, has greater efficacy, and induces fewer side effects [12].

In 1988, zolpidem was marketed in China as an effective drug for the short-term treatment of insomnia. Zolpidem is an imidazopyridine compound that selectively binds to the benzodiazepine ω1 receptor, which exerts inhibitory effects by partially activating GABA receptors (a ligand-gated ion channel) [13]; it exerts sedative and hypnotic effects but can easily lead to the development of drug tolerance and dependence. Due to its short peak latency and short half-life, it induces rapid sleep in insomnia patients and also reduces wakefulness after sleep onset (WASO), which is useful for the short-term treatment of insomnia [14].

However, individuals taking eszopiclone, zopiclone or zolpidem experience psychiatric distress, hangover-like symptoms, or even respiratory depression the next day in addition to the risks of developing drug tolerance and dependence [15]. Effective sleep aids should maximize perceived sleep quality and avoid drug-related adverse effects without changing the underlying sleep architecture [16]. Orexin is a neuropeptide produced by the ventrolateral hypothalamus (LH). Its isoforms (orexin A and orexin B) regulate the sleep-wake cycle [17]. Thus, orexin antagonists have been used to treat insomnia; suppressing excessive wakefulness and inducing and maintaining sleep by inhibiting the orexin signaling system [18,19]. The dual-receptor orexin antagonist (DORA) is a novel hypnotic drug; although animal experiments indicate slight cognitive facilitation of taking low doses of DORA, there have not been clinical studies or meta-analyses to determine whether DORA alters cognitive function in human subjects [20,21].

To date, no network meta-analysis comprehensively comparing the effects and applicability of commonly used hypnotics as well as hypnotic-induced cognitive impairments has been conducted. To provide a reference for the use of hypnotics, we categorized administered doses of four commonly used hypnotics as low, medium, or high doses and performed a comprehensive analysis of their efficacy and safety in terms of sleep, cognition, and adverse reactions.

## 2 Methods

This meta-analysis was guided by the Preferred Reporting Items for Systematic Reviews and Meta-analysis (PRISMA) guidelines [22]. The review scheme was registered with the International Prospective System Review Register (PROSPERO) (unique identifier: CRD42022352911).

### 2.1 Search strategy

The literature was retrieved from independent databases, including Google Scholar and the PubMed, Embase, Scopus, and Cochrane Library databases. The MeSH terms were (“dual orexin receptor antagonist” OR “suvorexant” OR “filorexant” OR “lemborexant” OR “almorexant” OR “daridorexant” OR “sb 649868”) AND (zolpidem) AND (eszopiclone) AND (zopiclone) AND (“Cognitive function” OR “cognitive function” OR “cognitive functions” OR “cognitional function” OR “Cognitive functioning” OR “Cognition function").

### 2.2 Inclusion and exclusion criteria

Inclusion criteria:

(1) Studies that included participants aged 18 years or older with or without insomnia and without other underlying diseases and mental disorders, such as depression and anxiety.
(2) Studies that compared the effects of DORA, zopiclone, eszopiclone, and zolpidem with those of the placebo (control group).
(3) Studies that reported at least one cognitive outcome, either scores on the Digit Symbol Substitution Test (DSST) or the daytime alertness test.
(4) Studies with a parallel, crossover or cohort design.

Exclusion criteria:

(1) Studies in which the treatment group(s) were treated with DORA, zopiclone, eszopiclone, and zolpidem in combination with other drugs;
(2) Repeat publications;
(3) Research with data that cannot be extracted (e.g., conference presentations) or are missing;
(4) Non-RCT publications, such as network meta-analyses, meta-analyses, systematic reviews, reviews, theoretical papers, methodological papers, animal experiments, case‒control studies and cohort studies.

### 2.3 Literature screening and data extraction

After searching for relevant records, references were extracted according to the search strategy. Endnote X9 software was used to eliminate duplicate records consistent with the inclusion standards. Relevant full texts were downloaded to determine whether they were eligible (i.e., met the inclusion standards). Two authors independently screened, extracted and cross-checked the selected records according to these standards. Disagreements were resolved through discussion with a third investigator. The data extracted were as follows: first author, publication date, sample size, sex ratio of participants, mean age of participants, mean disease duration, number of cases, intervention measures in the treatment group, intervention measures in the control group, course of treatment, outcome measures, and adverse reactions.

### 2.4 Bias risk assessment

Using the Risk of Bias tool in Review Manager 5.3 software, two evaluators independently evaluated the quality of each study from seven perspectives. Disagreements were resolved by discussion with the third investigator. Review Manager 5.3 software was used to visualize the results of the bias risk assessment for the literature included.

### 2.5 Statistical methods

Stata v.16.0 was applied. The standard mean difference (SMD) was generated as the effect size for continuous variables. Odds ratios (ORs) were produced for categorical variables. If only figures were provided, two researchers independently used GetData 2.26 to obtain the data and calculate the means. In cases where *I*^*2*^≤50 % and *p* > 0.01, the fixed effect model was applied. Otherwise, the random effect model was used. If *I*^2^>75 %, Galbraith plots were drawn to exclude studies outside the outlines to eliminate heterogeneity. Publication bias was evaluated by funnel plots and Egger's test. A *p* value < 0.05 was considered statistically significant.

## 3 Results

### 3.1 Study search and baseline characteristics and quality

**Fig. 1:**
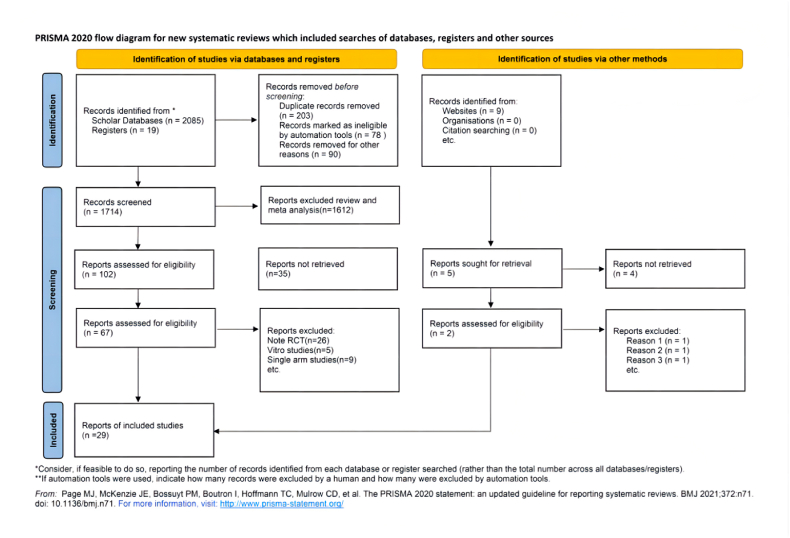
Preferred Reporting Items for Systematic Reviews and Meta-Analyses (PRISMA) flow diagram of study search and selection for the meta-analysis.

In the preliminary phase, a total of 2085 articles were searched, after scrupulous screening, eventually, 27 RCTs and 2 case-control studies were eligible for analysis (Fig. 1) [[23], [24], [25], [26], [27], [28], [29], [30], [31], [32], [33], [34], [35], [36], [37], [38], [39], [40], [41], [42], [43], [44], [45], [46], [47], [48], [49], [50], [51]]. The subjects came from America, Australia, British, and Germany, etc., their age mean ranged from 18 to 85, the general proportion of female was 47.3 %.

The DORA agents including Suvorexant, Filorexant, Lemborexant, Almorexant, Daridorexant, SB-649868. According to ROB2, all the 27 RCTs were with high quality, the 2 case-control study were with NOS score 8.

### 3.2 Digit Symbol Substitution Test (DSST)

**Fig. 2:**
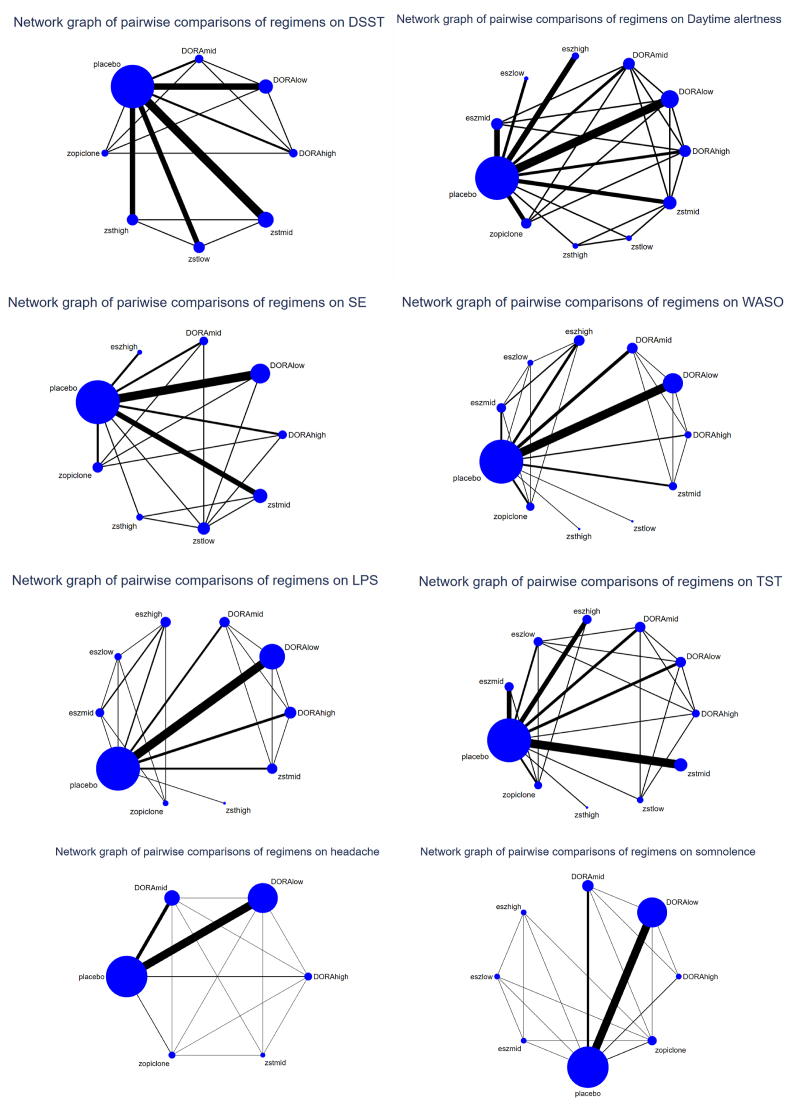
Network relationship diagram. The larger the circle, the more treatment measures were used. Thicker lines between two points indicate more studies including both topics.

No significant DSST results were obtained (see Fig. 1, Fig. 2, Table 1).

**Table 1:** Matrix of pairwise comparisons of regimens on DSST (shown as standard mean difference and 95 % confidence intervals).

	zstmid	DORAlow	placebo	DORAmid	DORAhigh	zstlow	zopiclone	zsthigh
SUCRA(%)	68.5	63.8	52.3	48.4	46.1	41.9	41	38.2
zstmid	0	0.15 (−3.11,3.42)	−0.34 (−1.25,0.58)	−0.43 (−3.71,2.84)	−0.25 (−4.68,4.17)	−0.57 (−2.05,0.91)	−0.17 (−3.43,3.10)	−0.65 (−2.13,0.83)
DORAlow	−0.15 (−3.42,3.11)	0	−0.49 (−3.62,2.64)	−0.59 (−4.22,3.04)	−0.62 (−3.18,1.94)	−0.73 (−4.07,2.61)	−0.32 (−3.94,3.30)	−0.80 (−4.14,2.54)
placebo	0.34 (−0.58,1.25)	0.49 (−2.64,3.62)	0	−0.10 (−3.24,3.05)	−0.03 (−2.61,2.54)	−0.23 (−1.40,0.93)	0.17 (−2.96,3.31)	−0.31 (−1.47,0.85)
DORAmid	0.43 (−2.84,3.71)	0.59 (−3.04,4.22)	0.10 (−3.05,3.24)	0	−0.12 (−4.56,4.32)	−0.14 (−3.49,3.21)	0.27 (−3.36,3.90)	−0.21 (−3.57,3.14)
DORAhigh	0.25 (−4.17,4.68)	0.62 (−1.94,3.18)	0.03 (−2.54,2.61)	0.12 (−4.32,4.56)	0	0.13 (−1.68,1.93)	−0.56 (−3.86,2.74)	−0.04 (−3.66,3.59)
zstlow	0.57 (−0.91,2.05)	0.73 (−2.61,4.07)	0.23 (−0.93,1.40)	0.14 (−3.21,3.49)	−0.13 (−1.93,1.68)	0	0.41 (−2.94,3.75)	−0.08 (−1.72,1.57)
zopiclone	0.17 (−3.10,3.43)	0.32 (−3.30,3.94)	−0.17 (−3.31,2.96)	−0.27 (−3.90,3.36)	0.56 (−2.74,3.86)	−0.41 (−3.75,2.94)	0	−0.48 (−3.83,2.86)
zsthigh	0.65 (−0.83,2.13)	0.80 (−2.54,4.14)	0.31 (−0.85,1.47)	0.21 (−3.14,3.57)	0.04 (−3.59,3.66)	0.08 (−1.57,1.72)	0.48 (−2.86,3.83)	0

### 3.3 Daytime alertness

Below, groups are referred to according to the drug and dose (e.g., eszopiclone_high_ indicates groups administered a high dose of eszopiclone). Eszopiclone_high_ had a significantly higher daytime alertness score (SMD = 3.00, 95 % CI: 1.86 to 4.13) than the placebo and a significantly higher daytime alertness score than (SMD = 4.21, 95 % CI: 1.65 to 6.77; SMD = 3.95, 95 % CI: 1.38 to 6.51; SMD = 3.26, 95 % CI: 0.38 to 6.15; and SMD = 3.23, 95 % CI: 0.34 to 6.11, respectively). DORA_mid_, compared with eszopiclone_high_, zolpidem_mid_ and eszopiclone_low_, had significantly lower daytime alertness scores (SMD = −3.16, 95 % CI: −6.04 to −0.27; SMD = −3.13, 95 % CI: −6.01 to −0.24; and SMD = −2.33, 95 % CI: −4.23 to −0.42, respectively), as shown in Table 2.

**Table 2:** Matrix of pairwise comparisons of regimens on Daytime alertness (shown as standard mean difference and 95 % confidence intervals).

	zstlow	zsthigh	DORAlow	eszmid	DORAmid	zstmid	DORAhigh	placebo	zopiclone	eszlow	eszhigh
SUCRA(%)	78.7	73.1	57.8	56.4	54.7	54.5	51	51	40.2	31.9	0.7
zstlow	0	0.27 (−2.98,3.51)	0.95 (−2.56,4.45)	0.99 (−2.52,4.49)	1.06 (−2.45,4.56)	1.09 (−2.42,4.59)	0.31 (−2.49,3.10)	1.21 (−1.08,3.51)	1.60 (−1.93,5.13)	1.88 (−0.87,4.64)	4.21 (1.65,6.77)
zsthigh	−0.27 (−3.51,2.98)	0	0.68 (−2.82,4.19)	0.72 (−2.78,4.23)	0.79 (−2.72,4.30)	0.82 (−2.68,4.33)	0.21 (−1.95,2.37)	0.95 (−1.35,3.24)	1.34 (−2.19,4.87)	1.62 (−1.14,4.38)	3.95 (1.38,6.51)
DORAlow	−0.95 (−4.45,2.56)	−0.68 (−4.19,2.82)	0	0.04 (−3.02,3.10)	0.11 (−2.95,3.17)	0.14 (−2.92,3.20)	0.25 (−1.92,2.41)	0.27 (−2.38,2.91)	0.66 (−2.43,3.74)	0.94 (−2.12,3.99)	3.26 (0.38,6.15)
eszmid	−0.99 (−4.49,2.52)	−0.72 (−4.23,2.78)	−0.04 (−3.10,3.02)	0	0.07 (−2.99,3.13)	0.10 (−2.96,3.16)	−0.69 (−2.85,1.47)	0.23 (−2.42,2.88)	0.62 (−2.47,3.71)	0.90 (−2.16,3.95)	3.23 (0.34,6.11)
DORAmid	−1.06 (−4.56,2.45)	−0.79 (−4.30,2.72)	−0.11 (−3.17,2.95)	−0.07 (−3.13,2.99)	0	0.03 (−3.03,3.09)	−0.01 (−3.75,3.74)	0.16 (−2.49,2.81)	0.55 (−2.54,3.64)	0.83 (−2.23,3.89)	3.16 (0.27,6.04)
zstmid	−1.09 (−4.59,2.42)	−0.82 (−4.33,2.68)	−0.14 (−3.20,2.92)	−0.10 (−3.16,2.96)	−0.03 (−3.09,3.03)	0	0.25 (−2.81,3.31)	0.13 (−2.52,2.77)	0.52 (−2.57,3.60)	0.80 (−2.26,3.85)	3.13 (0.24,6.01)
DORAhigh	−0.31 (−3.10,2.49)	−0.21 (−2.37,1.95)	−0.25 (−2.41,1.92)	0.69 (−1.47,2.85)	0.01 (−3.74,3.75)	−0.25 (−3.31,2.81)	0	0.10 (−3.64,3.85)	0.05 (−3.70,3.79)	3.02 (1.11,4.92)	−0.14 (−2.30,2.02)
placebo	−1.21 (−3.51,1.08)	−0.95 (−3.24,1.35)	−0.27 (−2.91,2.38)	−0.23 (−2.88,2.42)	−0.16 (−2.81,2.49)	−0.13 (−2.77,2.52)	−0.10 (−3.85,3.64)	0	0.39 (−2.29,3.07)	0.67 (−0.86,2.20)	3.00 (1.86,4.13)
zopiclone	−1.60 (−5.13,1.93)	−1.34 (−4.87,2.19)	−0.66 (−3.74,2.43)	−0.62 (−3.71,2.47)	−0.55 (−3.64,2.54)	−0.52 (−3.60,2.57)	−0.05 (−3.79,3.70)	−0.39 (−3.07,2.29)	0	0.28 (−2.81,3.37)	2.61 (−0.31,5.52)
eszlow	−1.88 (−4.64,0.87)	−1.62 (−4.38,1.14)	−0.94 (−3.99,2.12)	−0.90 (−3.95,2.16)	−0.83 (−3.89,2.23)	−0.80 (−3.85,2.26)	−3.02 (−4.92,-1.11)	−0.67 (−2.20,0.86)	−0.28 (−3.37,2.81)	0	2.33 (0.42,4.23)
eszhigh	−4.21 (−6.77,-1.65)	−3.95 (−6.51,-1.38)	−3.26 (−6.15,-0.38)	−3.23 (−6.11,-0.34)	−3.16 (−6.04,-0.27)	−3.13 (−6.01,-0.24)	0.14 (−2.02,2.30)	−3.00 (−4.13,-1.86)	−2.61 (−5.52,0.31)	−2.33 (−4.23,-0.42)	0

### 3.4 Sleep results: sleep efficiency (SE)

Compared with DORA_low_, zolpidem_low_ had significantly increased sleep efficiency (SE) (SMD = 2.95, 95 % CI: 0.04 to 5.86). Compared with the placebo, zolpidem_low_ had significantly increased SE (SMD = 3.27, 95 % CI: 0.47 to 6.07). Compared with zolpidem_mid_, the placebo had significantly reduced SE (SMD = −1.31, 95 % CI: −2.60 to −0.03), as shown in Table 3.

**Table 3:** Matrix of pairwise comparisons of regimens on SE (shown as standard mean difference and 95 % confidence intervals).

SUCRA(%)	zstlow	zstmid	DORAhigh	zopiclone	DORAmid	zsthigh	DORAlow	eszhigh	placebo
	95.3	80.1	51.5	48.5	44.5	40.4	36.2	33.4	20.2
zstlow	0	−1.64 (−4.62,1.34)	0.53 (−1.72,2.78)	−1.68 (−4.92,1.55)	−2.73 (−5.95,0.49)	−2.86 (−6.42,0.71)	−2.95 (−5.86,-0.04)	−3.05 (−6.27,0.18)	−3.27 (−6.07,-0.47)
zstmid	1.64 (−1.34,4.62)	0	0.75 (−0.84,2.34)	−0.04 (−2.99,2.90)	−1.09 (−2.97,0.80)	−1.21 (−3.64,1.22)	−1.31 (−2.60,-0.03)	−1.40 (−3.29,0.48)	−1.63 (−2.65,-0.61)
DORAhigh	−0.53 (−2.78,1.72)	−0.75 (−2.34,0.84)	0	0.71 (−3.25,4.67)	1.70 (−2.00,5.39)	−0.21 (−2.46,2.03)	1.61 (−2.04,5.27)	−0.44 (−2.21,1.34)	0.24 (−3.67,4.15)
zopiclone	1.68 (−1.55,4.92)	0.04 (−2.90,2.99)	−0.71 (−4.67,3.25)	0	−1.05 (−4.23,2.14)	−1.17 (−4.71,2.37)	−1.27 (−4.14,1.60)	−1.36 (−4.55,1.83)	−1.59 (−4.35,1.18)
DORAmid	2.73 (−0.49,5.95)	1.09 (−0.80,2.97)	−1.70 (−5.39,2.00)	1.05 (−2.14,4.23)	0	−0.13 (−2.85,2.59)	−0.22 (−1.99,1.55)	−0.32 (−2.56,1.93)	−0.54 (−2.13,1.05)
zsthigh	2.86 (−0.71,6.42)	1.21 (−1.22,3.64)	0.21 (−2.03,2.46)	1.17 (−2.37,4.71)	0.13 (−2.59,2.85)	0	−0.10 (−2.44,2.24)	−0.19 (−2.91,2.53)	−0.42 (−2.62,1.79)
DORAlow	2.95 (0.04,5.86)	1.31 (0.03,2.60)	−1.61 (−5.27,2.04)	1.27 (−1.60,4.14)	0.22 (−1.55,1.99)	0.10 (−2.24,2.44)	0	−0.09 (−1.86,1.68)	−0.32 (−1.10,0.47)
eszhigh	3.05 (−0.18,6.27)	1.40 (−0.48,3.29)	0.44 (−1.34,2.21)	1.36 (−1.83,4.55)	0.32 (−1.93,2.56)	0.19 (−2.53,2.91)	0.09 (−1.68,1.86)	0	−0.23 (−1.82,1.37)
placebo	3.27 (0.47,6.07)	1.63 (0.61,2.65)	−0.24 (−4.15,3.67)	1.59 (−1.18,4.35)	0.54 (−1.05,2.13)	0.42 (−1.79,2.62)	0.32 (−0.47,1.10)	0.23 (−1.37,1.82)	0

### 3.5 Sleep results: wakefulness after sleep onset (WASO)

Compared with the placebo, zolpidem_mid_ and DORA_high_ significantly reduced WASO (SMD = −2.83, 95 % CI: −4.84 to −0.83 and SMD = −4.48, 95 % CI: −7.14 to −1.81, respectively), whereas eszopiclone_low_ and zopiclone significantly increased WASO (SMD = 1.81, 95 % CI: 0.19 to 3.43 and SMD = 3.48, 95 % CI: 1.50 to 5.46, respectively). Compared with zolpidem_mid_, zolpidem_high_, eszopiclone_high_, eszopiclone_low_, and zopiclone significantly reduced WASO (SMD = −2.81, 95 % CI: −5.44 to −0.19; SMD = −2.75, 95 % CI: −4.92 to −0.59; SMD = −4.65, 95 % CI: −7.22 to −2.07; and SMD = −6.32, 95 % CI: −9.14 to −3.50, respectively). Compared with zolpidem_mid_, DORA_low_, DORA_mid_, eszopiclone_mid_, and eszopiclone_low_ significantly increased WASO (SMD = 4.65, 95 % CI: 2.07 to 7.22; SMD = 3.50, 95 % CI: 0.97 to 6.04; SMD = 3.48, 95 % CI: 0.94 to 6.02; and SMD = 2.63, 95 % CI: 0.77 to 4.49, respectively), see Table 4.

**Table 4:** Matrix of pairwise comparisons of regimens on WASO (shown as standard mean difference and 95 % confidence intervals).

SUCRA(%)	zstmid	DORAlow	DORAmid	eszmid	DORAhigh	zstlow	zsthigh	eszhigh	placebo	eszlow	zopiclone
	95.9	80.3	80.1	65.0	52.0	46.9	41.3	40.5	36.3	10.7	1.1
zstmid	0	1.14 (−1.14,3.43)	1.17 (−1.12,3.46)	2.01 (−0.19,4.22)	−0.28 (−2.73,2.18)	2.60 (−0.04,5.24)	2.81 (0.19,5.44)	2.75 (0.59,4.92)	2.83 (0.83,4.84)	4.65 (2.07,7.22)	6.32 (3.50,9.14)
DORAlow	−1.14 (−3.43,1.14)	0	0.02 (−2.21,2.26)	0.87 (−1.28,3.02)	1.22 (−0.36,2.80)	1.46 (−1.14,4.05)	1.67 (−0.91,4.25)	1.61 (−0.50,3.72)	1.69 (−0.25,3.64)	3.50 (0.97,6.04)	5.17 (2.40,7.95)
DORAmid	−1.17 (−3.46,1.12)	−0.02 (−2.26,2.21)	0	0.85 (−1.31,3.00)	−0.19 (−2.92,2.55)	1.43 (−1.16,4.03)	1.65 (−0.94,4.23)	1.59 (−0.53,3.70)	1.67 (−0.28,3.62)	3.48 (0.94,6.02)	5.15 (2.37,7.93)
eszmid	−2.01 (−4.22,0.19)	−0.87 (−3.02,1.28)	−0.85 (−3.00,1.31)	0	−3.38 (−6.43,-0.32)	0.59 (−1.36,2.53)	0.80 (−1.13,2.72)	0.74 (−0.49,1.96)	0.82 (−0.09,1.73)	2.63 (0.77,4.49)	4.30 (2.12,6.49)
DORAhigh	0.28 (−2.18,2.73)	−1.22 (−2.80,0.36)	0.19 (−2.55,2.92)	3.38 (0.32,6.43)	0	5.22 (3.53,6.92)	4.14 (1.43,6.85)	−1.20 (−2.78,0.39)	4.48 (1.81,7.14)	0.39 (−1.01,1.79)	2.28 (0.30,4.26)
zstlow	−2.60 (−5.24,0.04)	−1.46 (−4.05,1.14)	−1.43 (−4.03,1.16)	−0.59 (−2.53,1.36)	−5.22 (−6.92,-3.53)	0	0.21 (−2.20,2.62)	0.15 (−1.75,2.05)	0.23 (−1.48,1.95)	2.05 (−0.32,4.41)	3.72 (1.10,6.34)
zsthigh	−2.81 (−5.44,-0.19)	−1.67 (−4.25,0.91)	−1.65 (−4.23,0.94)	−0.80 (−2.72,1.13)	−4.14 (−6.85,-1.43)	−0.21 (−2.62,2.20)	0	−0.06 (−1.94,1.82)	0.02 (−1.67,1.72)	1.83 (−0.51,4.18)	3.51 (0.90,6.11)
eszhigh	−2.75 (−4.92,-0.59)	−1.61 (−3.72,0.50)	−1.59 (−3.70,0.53)	−0.74 (−1.96,0.49)	1.20 (−0.39,2.78)	−0.15 (−2.05,1.75)	0.06 (−1.82,1.94)	0	0.08 (−0.73,0.90)	1.89 (0.08,3.71)	3.56 (1.76,5.37)
placebo	−2.83 (−4.84,-0.83)	−1.69 (−3.64,0.25)	−1.67 (−3.62,0.28)	−0.82 (−1.73,0.09)	−4.48 (−7.14,-1.81)	−0.23 (−1.95,1.48)	−0.02 (−1.72,1.67)	−0.08 (−0.90,0.73)	0	1.81 (0.19,3.43)	3.48 (1.50,5.46)
eszlow	−4.65 (−7.22,-2.07)	−3.50 (−6.04,-0.97)	−3.48 (−6.02,-0.94)	−2.63 (−4.49,-0.77)	−0.39 (−1.79,1.01)	−2.05 (−4.41,0.32)	−1.83 (−4.18,0.51)	−1.89 (−3.71,-0.08)	−1.81 (−3.43,-0.19)	0	1.67 (−0.89,4.23)
zopiclone	−6.32 (−9.14,-3.50)	−5.17 (−7.95,-2.40)	−5.15 (−7.93,-2.37)	−4.30 (−6.49,-2.12)	−2.28 (−4.26,-0.30)	−3.72 (−6.34,-1.10)	−3.51 (−6.11,-0.90)	−3.56 (−5.37,-1.76)	−3.48 (−5.46,-1.50)	−1.67 (−4.23,0.89)	0

### 3.6 Sleep results: latency to persistent sleep (LPS)

Compared with the placebo, zolpidem_mid_, DORA_low_, and DORA_mid_ significantly reduced the latency to persistent sleep (LPS) (SMD = −1.14, 95 % CI: −2.02 to −0.26; SMD = −1.60, 95 % CI: −2.20 to −1.01; and SMD = −1.58, 95 % CI: −2.19 to −0.96, respectively). Compared with the placebo, eszopiclone_high_, eszopiclone_mid_, and DORA_high_ significantly reduced the LPS (SMD = −0.53, 95 % CI: −0.93 to −0.14; SMD = −0.52, 95 % CI: −1.01 to −0.02; and SMD = −6.25, 95 % CI: −7.67 to −4.84, respectively). Compared with the placebo, the LPS was significantly increased by (SMD = 3.03, 95 % CI: 1.94 to 4.12). Compared with the placebo, the LPS was significantly decreased by eszopiclone_high_, eszopiclone_mid_, and DORA_low_ (SMD = −1.07, 95 % CI: −1.78 to −0.35; SMD = −1.08, 95 % CI: −1.86 to −0.31; SMD = −1.18, 95 % CI: −1.96 to −0.41; and SMD = −1.22, 95 % CI: −1.74 to −0.70, respectively). Compared with eszopiclone_high_, eszopiclone_mid_, and DORA_low_, the LPS was significantly reduced by (SMD = −1.04, 95 % CI: −1.78 to −0.31; SMD = −1.06, 95 % CI: −1.85 to −0.27; and SMD = −1.16, 95 % CI: −1.95 to −0.37, respectively). Compared with zolpidem_mid_, DORA_low,_ DORA_mid_, and zopiclone significantly increased the LPS (SMD = 5.77, 95 % CI: 4.43 to 7.11; SMD = 4.63, 95 % CI: 3.38 to 5.88; and SMD = 4.61, 95 % CI: 3.35 to 5.86, respectively), as shown in Table 5.

**Table 5:** Matrix of pairwise comparisons of regimens on LPS (shown as standard mean difference and 95 % confidence intervals).

SUCRA(%)	zstmid	DORAlow	DORAmid	placebo	eszhigh	eszmid	eszlow	DORAhigh	zsthigh	zopiclone
	99.9	83.4	78.8	70.8	40.7	39.1	32.5	29.3	25.5	0.0
zstmid	0	1.14 (0.26,2.02)	1.17 (0.27,2.06)	2.74 (1.97,3.51)	2.21 (1.34,3.08)	2.23 (1.31,3.14)	2.33 (1.41,3.24)	−4.47 (−5.28,-3.65)	2.43 (1.54,3.33)	5.77 (4.43,7.11)
DORAlow	−1.14 (−2.02,-0.26)	0	0.02 (−0.72,0.77)	1.60 (1.01,2.20)	1.07 (0.35,1.78)	1.08 (0.31,1.86)	1.18 (0.41,1.96)	1.22 (0.70,1.74)	1.29 (0.54,2.04)	4.63 (3.38,5.88)
DORAmid	−1.17 (−2.06,-0.27)	−0.02 (−0.77,0.72)	0	1.58 (0.96,2.19)	1.04 (0.31,1.78)	1.06 (0.27,1.85)	1.16 (0.37,1.95)	−0.19 (−1.09,0.71)	1.27 (0.50,2.03)	4.61 (3.35,5.86)
placebo	−2.74 (−3.51,-1.97)	−1.60 (−2.20,-1.01)	−1.58 (−2.19,-0.96)	0	−0.53 (−0.93,-0.14)	−0.52 (−1.01,-0.02)	−0.42 (−0.91,0.08)	−6.25 (−7.67,-4.84)	−0.31 (−0.77,0.15)	3.03 (1.94,4.12)
eszhigh	−2.21 (−3.08,-1.34)	−1.07 (−1.78,-0.35)	−1.04 (−1.78,-0.31)	0.53 (0.14,0.93)	0	0.02 (−0.62,0.65)	0.12 (−0.52,0.75)	1.20 (0.66,1.74)	0.23 (−0.38,0.83)	3.56 (2.55,4.58)
eszmid	−2.23 (−3.14,-1.31)	−1.08 (−1.86,-0.31)	−1.06 (−1.85,-0.27)	0.52 (0.02,1.01)	−0.02 (−0.65,0.62)	0	0.10 (−0.60,0.80)	−5.15 (−6.57,-3.74)	0.21 (−0.47,0.89)	3.55 (2.35,4.75)
eszlow	−2.33 (−3.24,-1.41)	−1.18 (−1.96,-0.41)	−1.16 (−1.95,-0.37)	0.42 (−0.08,0.91)	−0.12 (−0.75,0.52)	−0.10 (−0.80,0.60)	0	0.16 (−0.34,0.65)	0.11 (−0.57,0.78)	3.45 (2.25,4.65)
DORAhigh	4.47 (3.65,5.28)	−1.22 (−1.74,-0.70)	0.19 (−0.71,1.09)	6.25 (4.84,7.67)	−1.20 (−1.74,-0.66)	5.15 (3.74,6.57)	−0.16 (−0.65,0.34)	0	3.38 (2.04,4.73)	−0.04 (−0.61,0.54)
zsthigh	−2.43 (−3.33,-1.54)	−1.29 (−2.04,-0.54)	−1.27 (−2.03,-0.50)	0.31 (−0.15,0.77)	−0.23 (−0.83,0.38)	−0.21 (−0.89,0.47)	−0.11 (−0.78,0.57)	−3.38 (−4.73,-2.04)	0	3.34 (2.15,4.53)
zopiclone	−5.77 (−7.11,-4.43)	−4.63 (−5.88,-3.38)	−4.61 (−5.86,-3.35)	−3.03 (−4.12,-1.94)	−3.56 (−4.58,-2.55)	−3.55 (−4.75,-2.35)	−3.45 (−4.65,-2.25)	0.04 (−0.54,0.61)	−3.34 (−4.53,-2.15)	0

### 3.7 Sleep results: total sleep time (TST)

Compared with the placebo, eszopiclone_mid_, zopiclone, zolpidem_mid_, and eszopiclone_high_ significantly increased the total sleep time (TST) (SMD = 2.39, 95 % CI: 1.11 to 3.67; SMD = 6.00, 95 % CI: 2.73 to 9.27; SMD = 1.89, 95 % CI: 0.90 to 2.88; and SMD = 1.70, 95 % CI: 0.42 to 2.99, respectively). Compared with the placebo, DORA_high_, zolpidem_low_, and eszopiclone_low_ significantly reduced the TST (SMD = −13.55, 95 % CI: −19.50 to −7.60; SMD = −8.82, 95 % CI: −13.65 to −3.98; and SMD = −16.12, 95 % CI: −21.34 to −10.90, respectively).

Zopiclone significantly increased the TST compared with zolpidem_mid_, eszopiclone_high_, DORA_mid_, and DORA_low_ (SMD = 3.61, 95 % CI: 0.10 to 7.12; SMD = 4.11, 95 % CI: 0.69 to 7.52; SMD = 6.65, 95 % CI: 1.42 to 11.89; and SMD = 6.93, 95 % CI: 1.69 to 12.17, respectively). Zolpidem_low_, compared with eszopiclone_mid_, zopiclone, zolpidem_mid_, and zolpidem_high_, significantly reduced the TST (SMD = −11.21, 95 % CI: −16.21 to −6.21; SMD = −14.82, 95 % CI: −20.65 to −8.98; SMD = −10.71, 95 % CI: −15.64 to −5.78; and SMD = −10.52, 95 % CI: −16.19 to −4.86, respectively), as shown in Table 6.

**Table 6:** Matrix of pairwise comparisons of regimens on TST (shown as standard mean difference and 95 % confidence intervals).

SUCRA(%)	eszmid	zopiclone	zstmid	eszhigh	zsthigh	DORAhigh	DORAmid	placebo	DORAlow	zstlow	eszlow
	82.0	81.7	71.0	67.0	67.0	65.8	36.2	35.9	33.4	10.0	0.1
eszmid	0	3.61 (0.10,7.12)	−0.50 (−2.12,1.12)	−0.68 (−2.50,1.13)	−0.68 (−3.90,2.54)	−14.46 (−20.40,-8.51)	−3.05 (−7.34,1.25)	−2.39 (−3.67,-1.11)	−3.32 (−7.62,0.97)	−11.21 (−16.21,-6.21)	−18.51 (−23.88,-13.14)
zopiclone	−3.61 (−7.12,-0.10)	0	−4.11 (−7.52,-0.69)	−4.29 (−7.30,-1.29)	−4.29 (−8.70,0.11)	17.58 (13.24,21.91)	−6.65 (−11.89,-1.42)	−6.00 (−9.27,-2.73)	−6.93 (−12.17,-1.69)	−14.82 (−20.65,-8.98)	−22.12 (−28.28,-15.96)
zstmid	0.50 (−1.12,2.12)	4.11 (0.69,7.52)	0	−0.19 (−1.81,1.44)	−0.19 (−3.30,2.93)	1.33 (−3.09,5.75)	−2.55 (−6.76,1.67)	−1.89 (−2.88,-0.90)	−2.82 (−7.04,1.39)	−10.71 (−15.64,-5.78)	−18.01 (−23.32,-12.70)
eszhigh	0.68 (−1.13,2.50)	4.29 (1.29,7.30)	0.19 (−1.44,1.81)	0	0.00 (−3.22,3.22)	2.11 (−0.78,5.00)	−2.36 (−6.66,1.93)	−1.70 (−2.99,-0.42)	−2.64 (−6.93,1.66)	−10.52 (−15.53,-5.52)	−17.83 (−23.20,-12.45)
zsthigh	0.68 (−2.54,3.90)	4.29 (−0.11,8.70)	0.19 (−2.93,3.30)	−0.00 (−3.22,3.22)	0	−2.63 (−7.16,1.91)	−2.36 (−7.41,2.69)	−1.71 (−4.66,1.25)	−2.64 (−7.69,2.41)	−10.52 (−16.19,-4.86)	−17.83 (−23.82,-11.83)
DORAhigh	14.46 (8.51,20.40)	−17.58 (−21.91,-13.24)	−1.33 (−5.75,3.09)	−2.11 (−5.00,0.78)	2.63 (−1.91,7.16)	0	0.40 (−4.58,5.39)	13.55 (7.60,19.50)	−2.39 (−5.28,0.51)	0.93 (−2.24,4.11)	0.25 (−2.93,3.43)
DORAmid	3.05 (−1.25,7.34)	6.65 (1.42,11.89)	2.55 (−1.67,6.76)	2.36 (−1.93,6.66)	2.36 (−2.69,7.41)	−0.40 (−5.39,4.58)	0	0.66 (−3.44,4.75)	−0.27 (−4.36,3.81)	−8.16 (−12.99,-3.34)	−15.46 (−20.68,-10.25)
placebo	2.39 (1.11,3.67)	6.00 (2.73,9.27)	1.89 (0.90,2.88)	1.70 (0.42,2.99)	1.71 (−1.25,4.66)	−13.55 (−19.50,-7.60)	−0.66 (−4.75,3.44)	0	−0.93 (−5.03,3.17)	−8.82 (−13.65,-3.98)	−16.12 (−21.34,-10.90)
DORAlow	3.32 (−0.97,7.62)	6.93 (1.69,12.17)	2.82 (−1.39,7.04)	2.64 (−1.66,6.93)	2.64 (−2.41,7.69)	2.39 (−0.51,5.28)	0.27 (−3.81,4.36)	0.93 (−3.17,5.03)	0	−7.89 (−12.72,-3.06)	−15.19 (−20.40,-9.98)
zstlow	11.21 (6.21,16.21)	14.82 (8.98,20.65)	10.71 (5.78,15.64)	10.52 (5.52,15.53)	10.52 (4.86,16.19)	−0.93 (−4.11,2.24)	8.16 (3.34,12.99)	8.82 (3.98,13.65)	7.89 (3.06,12.72)	0	−7.30 (−13.11,-1.49)
eszlow	18.51 (13.14,23.88)	22.12 (15.96,28.28)	18.01 (12.70,23.32)	17.83 (12.45,23.20)	17.83 (11.83,23.82)	−0.25 (−3.43,2.93)	15.46 (10.25,20.68)	16.12 (10.90,21.34)	15.19 (9.98,20.40)	7.30 (1.49,13.11)	0

### 3.8 Headache

There were no significant differences in headache, as shown in Table 7.

**Table 7:** Matrix of pairwise comparisons of regimens on somnolence (shown as standard mean difference and 95 % confidence intervals).

SUCRA(%)	eszlow	placebo	eszmid	zopiclone	eszhigh	DORAlow	DORAhigh	DORAmid
	86.0	78.3	63.9	62.8	56.4	31.9	14.0	6.8
eszlow	0	0.69 (−1.85,3.23)	1.14 (−2.07,4.36)	3.57 (−0.37,7.52)	1.46 (−1.71,4.63)	2.92 (−0.69,6.52)	−0.95 (−4.80,2.90)	4.30 (0.79,7.81)
placebo	−0.69 (−3.23,1.85)	0	0.45 (−1.52,2.42)	2.88 (−0.13,5.89)	0.77 (−1.12,2.66)	2.22 (−0.33,4.77)	−0.95 (−5.49,3.59)	3.60 (1.18,6.03)
eszmid	−1.14 (−4.36,2.07)	−0.45 (−2.42,1.52)	0	2.43 (−1.17,6.03)	0.32 (−2.42,3.05)	1.77 (−1.45,5.00)	2.34 (−0.29,4.96)	3.15 (0.03,6.28)
zopiclone	−3.57 (−7.52,0.37)	−2.88 (−5.89,0.13)	−2.43 (−6.03,1.17)	0	−2.11 (−5.67,1.44)	−0.66 (−2.26,0.94)	−0.00 (−3.94,3.94)	0.72 (−2.16,3.61)
eszhigh	−1.46 (−4.63,1.71)	−0.77 (−2.66,1.12)	−0.32 (−3.05,2.42)	2.11 (−1.44,5.67)	0	1.45 (−1.72,4.63)	−0.50 (−2.10,1.10)	2.83 (−0.24,5.91)
DORAlow	−2.92 (−6.52,0.69)	−2.22 (−4.77,0.33)	−1.77 (−5.00,1.45)	0.66 (−0.94,2.26)	−1.45 (−4.63,1.72)	0	0.88 (−0.91,2.67)	1.38 (−1.02,3.78)
DORAhigh	0.95 (−2.90,4.80)	0.95 (−3.59,5.49)	−2.34 (−4.96,0.29)	0.00 (−3.94,3.94)	0.50 (−1.10,2.10)	−0.88 (−2.67,0.91)	0	0.00 (−2.92,2.92)
DORAmid	−4.30 (−7.81,-0.79)	−3.60 (−6.03,-1.18)	−3.15 (−6.28,-0.03)	−0.72 (−3.61,2.16)	−2.83 (−5.91,0.24)	−1.38 (−3.78,1.02)	−0.00 (−2.92,2.92)	0

### 3.9 Sleepiness

Compared with the placebo, DORA_mid_ significantly increased sleepiness (SMD = 3.60, 95 % CI: 1.18 to 6.03). Compared with DORA_mid_, eszopiclone_low_ significantly reduced sleepiness (SMD = −4.30, 95 % CI: −7.81 to −0.79). Compared with eszopiclone_mid_, DORA_mid_ significantly increased sleepiness (SMD = 3.15, 95 % CI: 0.03 to 6.28), as shown in Table 8.

**Table 8:** Matrix of pairwise comparisons of regimens on headache (shown as standard mean difference and 95 % confidence intervals).

SUCRA(%)	zopiclone	placebo	DORAhigh	DORAlow	DORAmid	zstmid
	99.2	72.9	48.5	29.7	28.5	21.3
zopiclone	0	−1.63 (−3.53,0.26)	−1.06 (−3.13,1.00)	−0.45 (−2.95,2.05)	−0.47 (−2.70,1.76)	−0.25 (−2.46,1.96)
placebo	1.63 (−0.26,3.53)	0	−0.55 (−2.02,0.93)	1.18 (−0.87,3.23)	1.16 (−0.56,2.88)	1.38 (−0.30,3.06)
DORAhigh	1.06 (−1.00,3.13)	0.55 (−0.93,2.02)	0	0.56 (−1.29,2.42)	−0.00 (−2.93,2.93)	1.03 (−0.72,2.78)
DORAlow	0.45 (−2.05,2.95)	−1.18 (−3.23,0.87)	−0.56 (−2.42,1.29)	0	−0.02 (−2.39,2.35)	0.20 (−2.14,2.55)
DORAmid	0.47 (−1.76,2.70)	−1.16 (−2.88,0.56)	0.00 (−2.93,2.93)	0.02 (−2.35,2.39)	0	0.22 (−1.84,2.28)
zstmid	0.25 (−1.96,2.46)	−1.38 (−3.06,0.30)	−1.03 (−2.78,0.72)	−0.20 (−2.55,2.14)	−0.22 (−2.28,1.84)	0

In summary, DSST scores did not differ according to hypnotic, but there was a small-sample effect in the comparison of the placebo and zolpidem_high_. Daytime alertness did not consistently differ according to hypnotic, but there was a small-sample effect in the comparison of the placebo and eszopiclone_low_. The SE results were consistent. The WASO results significantly differed, but there was a small-sample effect in the comparison of eszopiclone_low_ and eszopiclone_mid_. There were significant differences in the LPS. There were significant differences in the TST and a small-sample effect in the comparison of the placebo and zolpidem_mid_. There were no significant differences in headache or sleepiness.

## 4 Discussion

To the best of our knowledge, this is the first network meta-analysis of the effects of hypnotic agents on sleep and cognition.

The DSST is part of the revised Webster Adult Intelligence Scale and is often used to assess information processing, attention, and psychomotor performance [52,53]. We found that none of the hypnotic agents, at any dose, significantly impacted DSST scores.

Compared to the placebo, eszopiclone_high_ impaired daytime alertness (SMD = 3.00, 95 % CI: 1.86 to 4.13); thus, those taking this drug should be warned about impaired performance when operating heavy machinery. Compared with the placebo, zolpidem_low_ significantly increased SE (SMD = 3.27, 95 % CI: 0.47 to 6.07). Reductions in SE can lead to memory loss, loss of focus, and significant declines in learning and work efficiency. Compared to the placebo, zolpidem_mid_ and DORA_high_ significantly reduced WASO (SMD = −2.83, 95 % CI: −4.84 to −0.83 and SMD = −4.48, 95 % CI: −7.14 to −1.81, respectively). Compared to the placebo, eszopiclone_low_ and zopiclone significantly increased WASO (SMD = 1.81, 95 % CI: 0.19 to 3.43; SMD = 3.48, 95 % CI: 1.50 to 5.46). Difficulty falling asleep and increased WASO are common types of sleep disturbance. Compared to the placebo, zolpidem_mid_, DORA_low_, and DORA_mid_ significantly reduced the LPS (SMD = −1.14, 95 % CI: −2.02 to −0.26; SMD = −1.60, 95 % CI: −2.20 to −1.01; and SMD = −1.58, 95 % CI: −2.19 to −0.96, respectively). Compared to the placebo, eszopiclone_mid_, zopiclone, zolpidem_mid_, and eszopiclone_high_ significantly increased the TST (SMD = 2.39, 95 % CI: 1.11 to 3.67; SMD = 6.00, 95 % CI: 2.73 to 9.27; SMD = 1.89, 95 % CI: 0.90 to 2.88; and SMD = 1.70, 95 % CI: 0.42 to 2.99, respectively). These four drug/dose combinations thus effectively prolonged sleep duration, which is beneficial because insufficient sleep duration leads to decreased energy and physical strength. Compared to the placebo, DORA_mid_ significantly increased sleepiness (SMD = 3.60, 95 % CI: 1.18 to 6.03).

Despite numerous clinical studies on hypnotherapy for insomnia, however, there is no clear conclusion on which hypnotic is the best treatment. We believe that a traditional meta-analysis, restricted to 2-by-2 comparisons, cannot provide valid methodological support for selecting the best intervention for hypnotherapy. In contrast, a network meta-analysis allows the comparison of multiple interventions. Therefore, this network meta-analysis provides the first comparison of the efficacy and safety of different hypnotic aids for insomnia using a frequency-based network meta-analysis framework, with the aims of comprehensively comparing direct and indirect treatments and providing more credible evidence for the clinical treatment of insomnia.

Over the years, DORA has been developed into a very successful hypnotic drug. DORA inhibits the hyperactive reticular activating system in insomnia by blocking orexin signal transduction [54]. An ideal hypnotic would have the following effects: rapid induction of sleep, sleep maintenance throughout the night (providing sufficient sleep), and little residual effects (e.g., sleepiness) the next morning [55]. DORA provides advantages such as improving sleep induction, enhancing metabolic waste removal, and improving circulation in the glial lymphoid system [56]. In a follow-up survey, DORA was well tolerated without serious safety problems or rebound or withdrawal reactions [57]. Regarding the effect of DORA on cognitive performance, orexinergic neurons directly project to the hippocampus and thereby influence learning, memory, and cognitive performance, indicating that DORA treatment may be a potential strategy to reverse the early cognitive impairment of insomnia patients [58]. Nonbenzodiazepines, including zolpidem, zopiclone, and eszopiclone are less addictive, have fewer neuromuscular effects, and lead to less cognitive impairment than benzodiazepines [59]. Zolpidem and dexzopiclone, in particular, only agonize hypnotic receptors and do not exert off-target effects on receptors involved in muscle relaxation, anxiety, or cognitive performance. Thus, nonbenzodiazepines are less disruptive to normal sleep architecture, are safer, and lead to less daytime sleepiness and other adverse effects than benzodiazepines [60]. However, as a first-line therapeutic drug in clinical application, zolpidem does not have satisfactory cognitive results. In addition, there is no clear “gold standard” for measuring hypnotic-related cognitive impairment. Zolpidem leads to few sequelae and withdrawal symptoms, is less likely to lead to drug tolerance and dependence, and has a wide range of safety. However, when used with other central inhibitors, it can cause severe respiratory depression. Therefore, it is suitable for occasional and temporary insomnia. Short-term use of zolpidem as a hypnotic is known to have common adverse reactions such as hallucinations, excitement, nightmares, and depression. Zopiclone and eszopiclone are representatives of the third generation of sedative hypnotic drugs. They have high efficacy, few adverse reactions, rapid action and effectiveness up to 6 h, enabling patients to fall asleep quickly and maintain sufficient sleep depth. Long-term use does not lead to obvious drug resistance or rebound after drug withdrawal. The latest drug, dexzopiclone, is a dextro (r-) isomer of zopiclone and is 2 times more potent than the s-isomer (zopiclone) with reduced toxicity. In terms of the likelihood of addiction, benzodiazepines > zopiclone > zolpidem.

The use of DORA as a potential preventive, therapeutic or neuroprotective drug that downregulates the orexinergic system [61] can not only treat sleep disruption in insomnia patients but also slow neurodegeneration process and cognitive impairment due to sleep loss. In short, DORA may treat a new aspect of (mainly mild to moderate) insomnia by improving sleep and enhancing cognition.

## 5 Limitations

1. Studies on each individual outcome were scarce; therefore, the sample sizes were small. Small sample sizes can result in higher variability, namely, wider 95 % confidence intervals, which indicates less stability of our results.
2. Likewise, owing to the scarcity of studies for certain outcomes, we were unable to analyze an outcome evaluated by two articles even though I^2^ ≥ 75 %; for these two articles, we describe the results only.
3. We did not account for the duration of drug use. The efficacy of agents (such as zolpidem) in inducing sleep gradually diminishes over time; thus, there would be potential bias without adjusting for the treatment period. However, only a few studies reported this confounding factor.
4. One of the articles included in our meta-analysis was not an RCT, potentially yielding bias.

## 6 Conclusion

In brief, DORA is a promising agent due to both its efficacy in treating insomnia and optimal safety, as indicated by a lack of cognitive impairment. Further head-to -head clinical studies are needed to confirm our findings.

## Supplementary note

Articles have been scrutinized with a professional anti plagiarism literature detection system prior to publication. Article statistical methods have been approved by Shandong First Medical University Graduate School Biology. Statistical expert review. This is an open access article which, under the terms of the Creative Commons license (4.0) (attribution 1 noncommercial share identical by descent), permits others to edit, adapt, and expand on the original article for non-commercial purposes, provided that the article is read, downloaded, copied, transmitted, printed, retrieved, hyperlinked by any user, And building for indexing, to be used as input data to the software or for any other legitimate use.

## Author contribution

The first author MZ obtains, analyzes or interprets the data of the work; The second author RJ drafted works or made critical modifications to important knowledge content. MZ, RJ has made significant contributions to the conception or design of the work, and finally approved the version to be published, and agreed to be responsible for all aspects of the work to ensure that problems related to the accuracy or completeness of any part of the work are properly investigated and resolved.

## Ethical conflict

I and other authors declare that this article does not involve any ethical conflict.

## Data availability statement

Data openly available in a public repository.

## Declaration of competing interest

Article writing and subject research process, no conflict of interest, no ethical conflicts involved content.

## References

[1] Julie A., Dopheide P. (2020).

[2] Brownlow J.A., Miller K.E., Gehrman P.R. (2020). Insomnia and cognitive performance. Sleep Med Clin.

[3] Fragale J.E. (2021). The insomnia-addiction positive feedback loop: role of the orexin system. Front Neurol Neurosci.

[4] Kent B.A., Mistlberger R.E. (2017). Sleep and hippocampal neurogenesis: implications for Alzheimer's disease. Front Neuroendocrinol.

[5] Picton J.D., Marino A.B., Nealy K.L. (2018). Benzodiazepine use and cognitive decline in the elderly. Am J Health Syst Pharm.

[6] Billioti de Gage S. (2014). Benzodiazepine use and risk of Alzheimer's disease: case-control study. BMJ.

[7] Nielsen S. (2017). Benzodiazepines. Curr Top Behav Neurosci.

[8] He D. (2020). Biphasic feature of placebo response in primary insomnia: pooled analysis of data from randomized controlled clinical trials of orexin receptor antagonists. Sleep.

[9] Ye C. (2021). Berberine improves cognitive impairment by simultaneously impacting cerebral blood flow and beta-amyloid accumulation in an APP/tau/PS1 mouse model of alzheimer's disease. Cells.

[10] Wang C., Holtzman D.M. (2020). Bidirectional relationship between sleep and Alzheimer's disease: role of amyloid, tau, and other factors. Neuropsychopharmacology.

[11] Morin C.M., Benca R. (2012). Chronic insomnia. The Lancet.

[12] Westermeyer J., Carr T.M. (2020). Zolpidem-associated consequences: an updated literature review with case reports. J Nerv Ment Dis.

[13] Holm Kristin J., Karen L. (2000).

[14] Kim H. (2020). Zolpidem overutilisation among Korean patients with insomnia. J Sleep Res.

[15] Foda N.H., Ali S.M. (2012). Zolpidem tartrate. Profiles Drug Subst Excipients Relat Methodol.

[16] Toor B. (2021). Sleep, orexin and cognition. Front Neurol Neurosci.

[17] Zhao P. (2022). Orexin A peptidergic system: comparative sleep behavior, morphology and population in brains between wild type and Alzheimer's disease mice. Brain Struct Funct.

[18] Mehr J.B., Bilotti M.M., James M.H. (2021). Orexin (hypocretin) and addiction. Trends Neurosci.

[19] Sakurai T. (2013). Orexin deficiency and narcolepsy. Curr Opin Neurobiol.

[20] Hamuro A., Honda M., Wakaura Y. (2018). Suvorexant for the treatment of insomnia in patients with Alzheimer's disease. Aust N Z J Psychiatr.

[21] Tisdale R.K., Yamanaka A., Kilduff T.S. (2021). Animal models of narcolepsy and the hypocretin/orexin system: past, present, and future. Sleep.

[22] Page M.J. (2021). The PRISMA 2020 statement: an updated guideline for reporting systematic reviews. BMJ.

[23] Vermeeren A. (2019). On-the-road driving performance the morning after bedtime administration of lemborexant in healthy adult and elderly volunteers. Sleep.

[24] Vermeeren A. (2014). Residual effects of low-dose sublingual zolpidem on highway driving performance the morning after middle-of-the-night use. Sleep.

[25] Tek C. (2014). The impact of eszopiclone on sleep and cognition in patients with schizophrenia and insomnia: a double-blind, randomized, placebo-controlled trial. Schizophr Res.

[26] Connor K.M., Mahoney E., Jackson S., Hutzelmann J., Zhao X., Jia N., Snyder E., Snavely D., Michelson D., Roth T., Herring W.J. (2016).

[27] Spierings E.L., McAllister P.J., Bilchik T.R. (2015). Efficacy of treatment of insomnia in migraineurs with eszopiclone (Lunesta(R)) and its effect on total sleep time, headache frequency, and daytime functioning: a randomized, double-blind, placebo-controlled, parallel-group, pilot study. Cranio.

[28] Zammit G. (2020). Daridorexant, a new dual orexin receptor antagonist, in elderly subjects with insomnia disorder. Neurology.

[29] Sun H. (2013). Effects of suvorexant, an orexin receptor antagonist, on sleep parameters as measured by polysomnography in healthy men. Sleep.

[30] Bland H. (2021). Effects of bedtime dosing with suvorexant and zolpidem on balance and psychomotor performance in healthy elderly participants during the night and in the morning. J Clin Psychopharmacol.

[31] Louzada L.L. (2022). The efficacy and safety of zolpidem and zopiclone to treat insomnia in Alzheimer's disease: a randomized, triple-blind, placebo-controlled trial. Neuropsychopharmacology.

[32] Menza M. (2010). Treatment of insomnia in Parkinson's disease: a controlled trial of eszopiclone and placebo. Mov Disord.

[33] Karppa M. (2020). Long-term efficacy and tolerability of lemborexant compared with placebo in adults with insomnia disorder: results from the phase 3 randomized clinical trial SUNRISE 2. Sleep.

[34] Mets M.A. (2011). Next-day effects of ramelteon (8 mg), zopiclone (7.5 mg), and placebo on highway driving performance, memory functioning, psychomotor performance, and mood in healthy adult subjects. Sleep.

[35] Uchimura N., Kamijo A., Takase T. (2012). Effects of eszopiclone on safety, subjective measures of efficacy, and quality of life in elderly and nonelderly Japanese patients with chronic insomnia, both with and without comorbid psychiatric disorders: a 24-week, randomized, double-blind study. Ann Gen Psychiatr.

[36] Uchimura N. (2012). A randomized placebo-controlled polysomnographic study of eszopiclone in Japanese patients with primary insomnia. Sleep Med.

[37] Bettica P. (2012). The orexin antagonist SB-649868 promotes and maintains sleep in men with primary insomnia. Sleep.

[38] Murphy P. (2020). Safety of lemborexant versus placebo and zolpidem: effects on auditory awakening threshold, postural stability, and cognitive performance in healthy older participants in the middle of the night and upon morning awakening. J Clin Sleep Med.

[39] Murphy P. (2017). Lemborexant, A dual orexin receptor antagonist (DORA) for the treatment of insomnia disorder: results from a bayesian, adaptive, randomized, double-blind, placebo-controlled study. J Clin Sleep Med.

[40] Hoever P. (2012). Orexin receptor antagonism: an ascending multiple-dose study with almorexant. J Psychopharmacol.

[41] Hoever P. (2013). Tolerability, pharmacokinetics, and pharmacodynamics of single-dose almorexant, an orexin receptor antagonist, in healthy elderly subjects. J Clin Psychopharmacol.

[42] Sonia Ancoli-Israel P. (2010).

[43] Leufkens T.R., Lund J.S., Vermeeren A. (2009). Highway driving performance and cognitive functioning the morning after bedtime and middle-of-the-night use of gaboxadol, zopiclone and zolpidem. J Sleep Res.

[44] Leufkens T.R. (2014). Residual effects of zopiclone 7.5 mg on highway driving performance in insomnia patients and healthy controls: a placebo controlled crossover study. Psychopharmacology (Berl).

[45] Herring W.J. (2016). Suvorexant in patients with insomnia: results from two 3-month randomized controlled clinical trials. Biol Psychiatr.

[46] Herring W.J. (2017). Suvorexant in elderly patients with insomnia: pooled analyses of data from phase III randomized controlled clinical trials. Am J Geriatr Psychiatr.

[47] Herring W.J. (2013). Alertness and psychomotor performance effects of the histamine-3 inverse agonist MK-0249 in obstructive sleep apnea patients on continuous positive airway pressure therapy with excessive daytime sleepiness: a randomized adaptive crossover study. Sleep Med.

[48] Joseph Herring W., Snyder Ellen, Budd Kerry, Hutzelmann Jill, Snavely Duane, Liu Kenneth, Lines Christopher, Roth Thomas, Michelson David (2012).

[49] Herring W.J. (2016). Suvorexant in patients with insomnia: pooled analyses of three-month data from phase-3 randomized controlled clinical trials. J Clin Sleep Med.

[50] Dauvilliers Y. (2020). Daridorexant, a new dual orexin receptor antagonist to treat insomnia disorder. Ann Neurol.

[51] Zhang Y. (2018). Cognitive behavioral therapy for insomnia combined with eszopiclone for the treatment of sleep disorder patients transferred out of the intensive care unit: a single-centred retrospective observational study. Medicine (Baltimore).

[52] Zihl J. (2014). Cognitive reserve in young and old healthy subjects: differences and similarities in a testing-the-limits paradigm with DSST. PLoS One.

[53] Baune B.T., Brignone M., Larsen K.G. (2018). A network meta-analysis comparing effects of various antidepressant classes on the Digit Symbol substitution test (DSST) as a measure of cognitive dysfunction in patients with major depressive disorder. Int J Neuropsychopharmacol.

[54] Kawabe K. (2017). Suvorexant for the treatment of insomnia in adolescents. J Child Adolesc Psychopharmacol.

[55] Gentile T.A. (2018). Suvorexant, an orexin/hypocretin receptor antagonist, attenuates motivational and hedonic properties of cocaine. Addiction Biol.

[56] Kuriyama A., Tabata H. (2017). Suvorexant for the treatment of primary insomnia: a systematic review and meta-analysis. Sleep Med Rev.

[57] Bollu Pradeep C. (2019).

[58] Kusztor A. (2019). Sleep deprivation differentially affects subcomponents of cognitive control. Sleep.

[59] Olsson M. (2018). Sleep deprivation and cerebrospinal fluid biomarkers for Alzheimer's disease. Sleep.

[60] Bahureksa L. (2017). The impact of mild cognitive impairment on gait and balance: a systematic review and meta-analysis of studies using instrumented assessment. Gerontology.

[61] Kumar A., Chanana P., Choudhary S. (2016). Emerging role of orexin antagonists in insomnia therapeutics: an update on SORAs and DORAs. Pharmacol Rep.

